# Chondroitin Sulfate Proteoglycans in the Nervous System: Inhibitors to Repair

**DOI:** 10.1155/2014/845323

**Published:** 2014-09-18

**Authors:** Justin R. Siebert, Amanda Conta Steencken, Donna J. Osterhout

**Affiliations:** ^1^Lake Erie College of Osteopathic Medicine at Seton Hill, 20 Seton Hill Drive, Greensburg, PA 15601, USA; ^2^Department of Cell and Developmental Biology, State University of New York Upstate Medical University, 750 East Adams Street, Syracuse, NY 13210, USA

## Abstract

Chondroitin sulfate proteoglycans (CSPGs) are widely expressed in the normal central nervous system, serving as guidance cues during development and modulating synaptic connections in the adult. With injury or disease, an increase in CSPG expression is commonly observed close to lesioned areas. However, these CSPG deposits form a substantial barrier to regeneration and are largely responsible for the inability to repair damage in the brain and spinal cord. This review discusses the role of CSPGs as inhibitors, the role of inflammation in stimulating CSPG expression near site of injury, and therapeutic strategies for overcoming the inhibitory effects of CSPGs and creating an environment conducive to nerve regeneration.

## 1. Introduction

The limited ability of the human central nervous system (CNS) to repair itself following injuries has been known since the days of Ancient Egypt (3,000–2,5000 BCE), as documented in the Edwin Smith papyrus [1]. It had long been thought that neurons in the CNS were incapable of mounting a regenerative response, until the studies of Aguayo and colleagues in the early 1980's [2, 3] which demonstrated that certain classes of neurons within the CNS, particularly those neurons which sustained an axonal injury in close proximity to their cell body, were able to regenerate their axons within a permissive environment, such as a peripheral nerve graft. Aguayo's work and more recent studies [4–6] have all demonstrated that supraspinal neurons (neurons arising in the cerebral cortex or brainstem and which project their axons caudally into the spinal cord) are actually capable of mounting a regenerative, albeit brief, and response following injury, when provided with the proper environment. While advances in science have not solved the problem of this short and often abortive nature of CNS neuron regeneration, many of the studies point to the same general theme: CNS neurons attempt to regenerate, but the post-injury environment is highly inhibitory to this process due to many molecules expressed after damage to the nervous system. One family of molecules, the chondroitin sulfate proteoglycans (CSPGs), are of particular importance and have significant roles in limiting the reparative response in almost every case of CNS damage.

Injuries to the CNS can generally be classified into two overarching categories: traumatic and neurodegenerative. Traumatic lesions to the brain or spinal cord are largely contusive in nature and often result from falls, sharp blows, or sudden deceleration style injuries, rather than penetrating wounds [7, 8]. Unlike sharp lacerating wounds that sever tissue, contusion lesions occur when a physical force (compression, shearing, or tensile) is rapidly applied to neural tissue without cutting [7, 9–11]. These sudden forces cause rapid and focal compression and displacement of neural tissue, resulting in the disruption of multiple afferent and efferent neuronal fiber tracts. Nontraumatic injuries to the CNS are often caused by degenerative pathologies, such as multiple sclerosis, Alzheimer's disease, and Parkinson's disease. While research is progressing in all arenas of traumatic and degenerative CNS lesions, one common attribute is observed: the expression of CSPGs in and around the areas of CNS tissue damage. It is important to understand that upregulation of CSPG expression in response to an insult is thought to be a protective mechanism, an attempt to wall off the area of damage and limit its spread [12–15]. However, this creates a cellular microenvironment that inhibits regeneration and repair. It follows then that one therapeutic approach to enhance CNS repair involves modulation of CSPG expression, which can change the cellular environment to allow for neural regeneration.

## 2. Chondroitin Sulfate Proteoglycans

**Figure 1:**
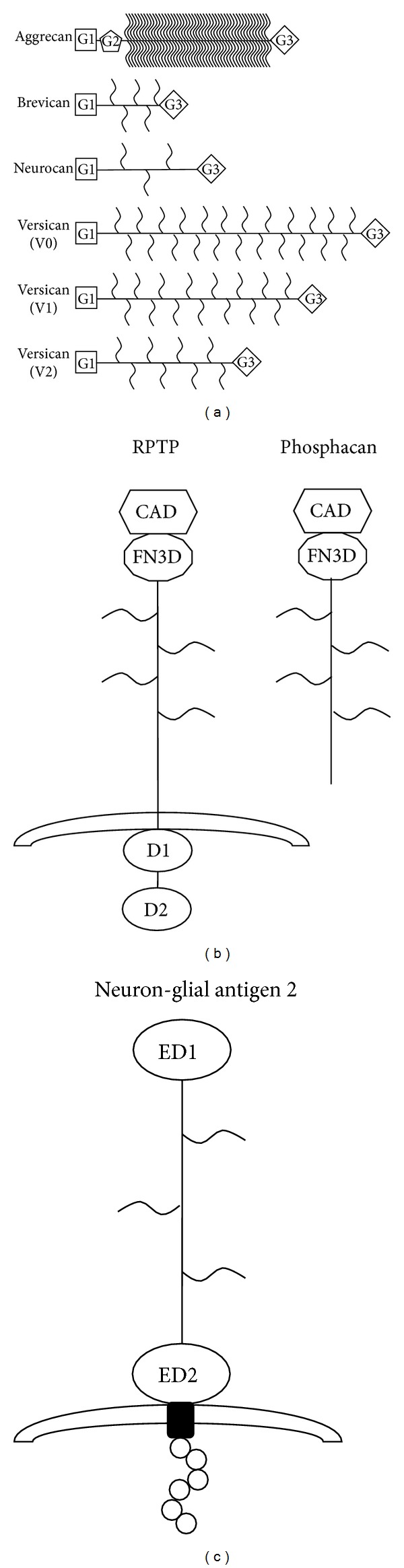
Schematic representation of individual proteoglycan molecules. (a) Members of the lectican family: aggrecan, brevican neurocan, and the three isotypes of versican, all share a similar homology, with a G1 domain at the N-terminus and a G3 domain at the C-terminus. The GAG side chain varies in number among the different lectican family members but is attached to the central core of the protein. (b) Phosphacan is a splice variant of the RPTP molecule, lacking the transmembrane and two intracellular domains, found in the RPTP molecule. (c) NG2 is a transmembrane proteoglycan that lacks homology to any of the other CSPGs. NG2 has two large extracellular domains separated by an extended region, where the GAGs are attached, a transmembrane domain and short cytoplasmic tail. NG2 can be cleaved by enzymes at the cell surface and released into the extracellular matrix (adapted and modified from [16, 18]).

Among the many CSPG molecules expressed in the CNS are the lectican group, which include aggrecan, three forms of versican (V0, V1, and V2), neurocan, and brevican (Figure 1). All members of the lectican family consist of a central core protein that has an N-terminal G1 domain and a C-terminal G3 domain. The central domain binds the chondroitin sulfate glycosaminoglycan side chains (CS-GAG) [16–18]. The aggrecan proteoglycan is the only member of the lectican group that has an additional globular (G2) near the G1 domain. Individual lectican molecules differ in the number of CS-GAG chains attached to their core proteins, with over one hundred GAG side chains being found in aggrecan and as little as zero to five GAG chains being found in brevican and neurocan [18] (Figure 1). The lectican family of CSPGs is largely produced by two major cell groups in the CNS: neurons and astrocytes (Table 1).

Other proteoglycans that have major roles in the pathology of CNS injury are phosphacan and NG2 (Figure 1). Phosphacan is a splice variant of receptor-type protein tyrosine phosphatase (RPTP) and lacks the two intracellular tyrosine phosphatase domains that are found in RPTP [18]. This RPTP splice variant is secreted into the extracellular environment and contains attachment regions for CS-GAG chains. Neuron-glial antigen 2 (NG2) is a unique CSPG that lacks sequence homology to other known CSPGs. NG2 proteoglycan is a transmembrane proteoglycan, composed of a large extracellular domain (two large globular domains separated by an extended region to which the GAGs attach), transmembrane domain, and a short cytoplasmic tail (Figure 1) [18, 19]. Cells that express the NG2 proteoglycan include oligodendrocyte progenitor cells, polydendrocytes, activated microglia, and activated macrophages (Table 1).

**Figure 2:**
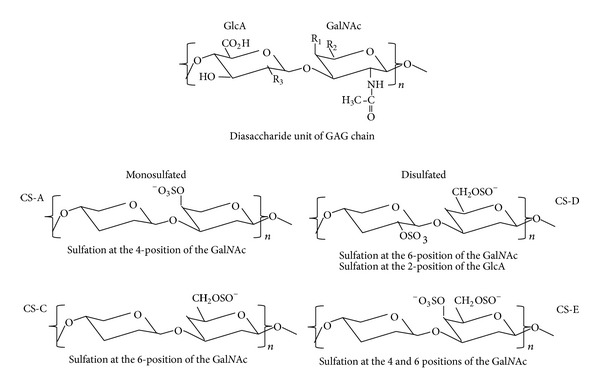
Diagram of the sulfation patterns of the disaccharide unit of the GAG chain. Sulfation at different carbon atom positions in the GlcA and/or Gal*N*Ac saccharide unit is one of the major factors that influence the effects of the proteoglycan. Monosulfation can occur at position 4 of the Gal*N*Ac resulting in synthesis of CS-A GAG or position 6 of the Gal*N*Ac resulting in the synthesis of the CS-C GAG. Disulfation can also occur with sulfation of position 6 of the Gal*N*Ac and position 2 of the GlcA, resulting in synthesis of the CS-D GAG, or sulfation of positions 4 and 6 on the Gal*N*Ac saccharide unit resulting in formation of the CS-E GAG (adapted and modified from [16, 18]).

The variations in the length of the core protein and the varying number of GAG side chains attached to the central domain are major determinants of the biological activity of an individual proteoglycan. However, the biological effects of a proteoglycan are also affected by the sulfation patterns of the* N*-acetylgalactosamine and glucuronate disaccharide, the basic unit that composes the CS-GAG [16, 18]. There are four different sulfation patterns that can occur, based on either monosulfation or disulfation of the* N*-acetylgalactosamine (Gal*N*Ac) and glucuronate disaccharide (GlcA) of the GAG disaccharide, leading to the synthesis of the CS-A, CS-C, CS-D, and CS-E CS-GAGs (Figure 2) [16–18, 20]. Sulfation of the chondroitin sulfate disaccharide can occur in the 4 and 6 positions of the Gal*N*Ac and the 2 position of the GlcA [16, 18, 20]. It has been demonstrated that a CSPG molecule can be either inhibitory or permissive for axonal growth, based on the molecular position of the sulfation which occurs in the disaccharide [20–22]. For example, when the sulfation occurs at the 4-position (CS-A), the proteoglycan tends to be highly inhibitory to axonal outgrowth [21, 22] whereas sulfation in the 6-position (CS-C) is more controversial and has been found to be inhibitory to axonal outgrowth by some labs [23] and permissive to axonal growth in others [20].* In vitro* experiments show that the disulphated GAGs (CS-D and CS-E) promote the growth of embryonic axons [24, 25]. Taken together, these observations reveal many aspects of proteoglycan structure which can modulate their biological effects on cells within the CNS.

The majority of CSPGs can interact with growth factors, cell adhesion molecules, and other ECM molecules in the local environment and may regulate their biological activity. CSPGs are widely distributed throughout the normal CNS, both during development and in the adult. CSPGs expression is especially rich in the embryonic brain and can direct cell migration and axonal outgrowth by providing guidance cues [17, 18]. Phosphacan is concentrated in the regions of cell proliferation such as the ventricular zone of the embryonic brains, suggesting it may modulate cell division. In the healthy adult nervous system, the soma and proximal dendrites of certain neurons are surrounded by a CSPG rich structure known as a perineuronal net. This perineuronal net stabilizes existing synapses and inhibits the formation of aberrant synaptic connections [17, 26]. In terms of general CSPG expression patterns, white matter is rich in versican and neurocan, while brevican can be found throughout the CNS, and NG2 is found expressed among meningeal cells, blood vessels, and OPCs [27].

## 3. Traumatic Lesions

Vast strides have been made in characterizing and understanding the complex orchestration of biological events that occurs following a lesion to the CNS. Unfortunately, it is now widely known and accepted that the events occurring in brain or spinal cord tissue post-injury create an environment hostile to a regenerative response, resulting in the abortive nature of the reparative process [9, 28, 29]. This is due in part to a significant upregulation of CSPGs.

The extreme forces applied to the CNS during traumatic injuries result in disruption of axonal tracts, blood vessels, and glial cells located at the epicenter of the lesion (reviewed by [10, 12]). Immediately following injury, there is a marked swelling of CNS tissue caused by damage to the local vasculature, allowing for leakage of blood plasma fluid into the surrounding extracellular space [30]. This vascular damage also creates an anoxic environment that is accompanied by necrosis of tissue damaged during the injury. Dying cells release their contents directly into the extracellular environment, resulting in a massive infiltration of blood borne macrophages and microglia, the resident CNS immune cell [31–34].

In healthy CNS tissue, microglial cells exist in a nonactive or resting state, courtesy of microglia-neuronal communications [35–37]. However, with an infection or trauma, microglial cells are activated, taking on the role of a phagocytic macrophage [38]. Activated macrophages/microglia play an important role in the CNS response to injury and infection because of the various products they secrete [37], including cytotoxic molecules (free radicals, i.e., superoxides), neurotrophic molecules (nerve growth factor, e.g.), and a variety of pro- and anti-inflammatory cytokines and chemokines [37]. These cytokines can stimulate the expression of CSPGs in a variety of cells in and around the lesion. Complicit in this process is the recruitment of blood borne macrophages which invade the lesion site. As these cells perform their biological function of phagocytizing necrotic cellular debris and any foreign pathogens, they further exacerbate the inflammatory response by releasing inflammatory cytokines, which can also stimulate CSPGs synthesis from neighboring cells [38]. Most important are the astrocytes located at the border of the injury, which undergo a process known as reactive astrogliosis. In response to various cytokines, astrocytes become hypertrophic and begin secreting CSPGs, leading to the formation of a glial scar. The glial scar serves as a chemophysical barrier to axonal regeneration [32, 33, 39, 40].

## 4. Degenerative Lesions

While the etiologies of traumatic and degenerative lesions are different, during degeneration the CNS responds in a highly similar manner to that described for traumatic lesions, complete with reactive astrogliosis and the deposition of CSPGs. Hallmarks of Alzheimer's disease (AD) pathology include the formation of neurofibrillary tangles and extracellular accumulation of amyloid-beta (A*β*) fibrils [41]. This results in a loss of axonal integrity and a decline in synaptic connectivity that is believed to contribute to dementia [42]. Dystrophic neurites, activated astrocytes, and A*β* fibrils form senile plaques, which are the key diagnostic criteria of AD [43, 44]. These senile plaques appear to be loci for inflammatory processes, containing a variety of molecules such as cytokines, acute-phase proteins, and complement proteins, which are secreted by reactive microglia and astrocytes around the lesion [45–47]. Microglia have been identified in close association with AD lesions, and while their function is to phagocytize the lesions, they are unable to do so. With plaque deposition, activated microglia are present on a continuous basis and thus provide a constant source of neurotoxic molecules [48]. This mechanism has been proposed to explain the toxicity of amyloid peptides (reviewed by [49–53]).

Parkinson's disease (PD) is a neurodegenerative movement disorder of unknown etiology, characterized by the selective loss of dopaminergic nigrostriatal neurons [54]. PD patients present symptoms such as resting tremor, postural instability, muscle stiffness, and slowness of voluntary movements (reviewed by [55–57]). The selective vulnerability of dopaminergic neurons observed in PD is thought to be due to various interacting factors, one of which is the microglia-mediated inflammatory response [57, 58]. A role for activated microglia in neurodegeneration is supported by studies using PD animal models. Dopaminergic neuronal degeneration can be elicited by injecting the neurotoxin bacterial lipopolysaccharide into the rat substantia nigra. These experiments revealed a significant increase in microglia activation throughout this subcortical area preceding marked neuronal death [59]. Furthermore, lipopolysaccharide-induced neuronal death was blocked by inhibition of microglial activation in this model. Administration of the neurotoxins 1-Methyl-4-phenyl-1,2,5,6-tetrahydropyridine (MPTP) or 6-hydroxydopamine (6-OHDA) also mimics PD symptoms in mice and induces activation and proliferation of microglia, as well as increased expression of inducible nitric oxide synthase (iNOS) and MHC class-I and II molecules [57, 60–64]. The observed increase in activated microglia is highly localized and anatomically discrete, limited to the substantia nigra. Furthermore, it is directly correlated with the neuronal death (reviewed by [57]).

Multiple sclerosis (MS) is a clinically heterogeneous demyelinating disease of the CNS [65], characterized by inflammation, axonal degeneration, and gliosis [66]. The etiology of this chronic inflammatory disease is unknown, although an autoimmune response is thought to be involved. MS lesions are characterized by the presence of large regions of demyelination, commonly referred to as plaques. These plaques contain reactive glial scar formation, with infiltration of activated T cells and macrophages [67]. The blood brain barrier (BBB) is disrupted, and upregulation of various adhesion molecules has been reported on capillary endothelial cells during the early stages of disease [68]. Increased BBB permeability allows for inflammatory infiltrates like activated macrophages, T cells, and antibodies, to invade the CNS parenchyma. Tumor necrosis factor alpha (TNF-*α*) is a key cytokine that may influence the progression of MS. TNF-*α* promotes the proliferation of bovine astrocytes and human astroglioma cell lines, which leads to reactive gliosis, as seen in active MS plaques [69]. TNF-*α* also stimulates production of colony-stimulating factors from neighboring astrocytes, which act as chemoattractants for reactive cells. This results in increased migration of activated cells to sites of inflammation and increases in the proliferation and activation of microglia [70–72].

Amyotrophic lateral sclerosis (ALS) is a rapidly progressing, adult-onset disease that usually results in death within 5 years of its initiation. Clinical manifestations of the disease include initial muscle weakness and atrophy that progress to a spastic paralysis resulting from motor neuron degeneration [73]. About 25% of ALS cases appear to be caused by a gain-of-function mutation in the antioxidant enzyme Cu, Zn superoxide dismutase-1 (SOD-1), which catalyzes the conversion of superoxide to oxygen and hydrogen peroxide [74]. This disease shares some of the characteristic neuroinflammatory changes observed in other neurodegenerative diseases like AD and MS [75]. Neuroinflammation is a key mediator of the pathology observed in ALS [76]. Reactive microglia and macrophages have been detected in spinal cord and motor cortex of ALS patients using conventional immunohistochemistry for activated monocyte-lineage-cell antigens (reviewed by [75]). MHC-I and MHC-II molecules, *β*2-integrins, and leukocyte common antigen are upregulated, indicating the presence of reactive microglia in these tissues [75, 77, 78]. Activated astrocytes and leukocytes are also observed, as demonstrated by glial fibrillary acidic protein (GFAP) and leukocyte functional antigen (LFA)-1 staining, respectively. Furthermore, the majority of LFA-1^+^ leukocytes were CD8^+^ [79], suggesting a role for cytotoxic T lymphocytes in disease progression. This T cell presence is not as prominent as that seen in T cell-mediated diseases like MS. In contrast, it has been proposed that neuroinflammation in ALS and its animal models is driven mainly by reactive macrophages and microglia and the resulting dysregulation in cytokine expression [76].

## 5. Role of Inflammation in Stimulating CSPG Expression

Trauma-induced CNS injury and autoimmune/neurodegenerative CNS disorders have several important similarities and differences in terms of the immune/inflammatory response. This observation was noted by Popovich and colleagues [80] comparing MS and SCI, and it could easily be further extended to include conditions like ALS, AD, and PD. For example, one key feature observed in CNS diseases is the disruption of the BBB, which allows for an influx of inflammatory cells [81–84]. In AD, PD, MS, and ALS, the mechanisms for increased BBB permeability remain unknown. Inflammatory mediators released from microglia and T lymphocytes are related to this process, but what triggers this release is still a matter of controversy. On the other hand, in cases of traumatic CNS injuries, BBB disruption is a direct consequence of traumatic insult. Nonetheless, the chronic endothelial permeability is maintained and explained by a perpetuated intraparenchymal inflammatory response.

Both traumatic and autoimmune/neurodegenerative CNS injuries show microglial activation and immune cell infiltration. Even the temporal sequence of events is similar in these scenarios, where microglia are recruited first, releasing inflammatory cytokines and reactive oxygen species (ROS), as well as upregulating their antigen presenting cell capabilities. Following increased BBB permeability, hematogenous macrophages, neutrophils, and lymphocytes follow, mediating myelin vesiculation, lipid peroxidation and further release of proinflammatory agents and free radicals. Demyelination ensues, resulting from oligodendrocyte injury and edema. However, in SCI this process is restricted to CNS myelin, whereas in conditions like MS, myelin destruction and ROS are found in the CNS as well as in the periphery [80].

Increases in pro- and anti-inflammatory cytokine levels and ROS production are a common occurrence in traumatic and autoimmune or neurodegenerative CNS disorders. In terms of therapeutic approaches, this has been targeted repeatedly using a variety of strategies that include antibody treatments, administration of cytokine receptor antagonists, or even proinflammatory cytokines themselves [85–90]. Glucocorticoids like methylprednisolone have been used extensively in light of their ability to suppress proinflammatory cytokine synthesis [91]. Because activation of various immune cells during the inflammatory response is directly associated with secretion of ROS and tyrosine nitration, approaches such as antioxidant administration, inhibition of inducible nitric oxide synthase iNOS, and targeted depletion of hematogenous macrophages are all under investigation [92].

One of the main differences between SCI and autoimmune/neurodegenerative disorders is the induction time for cytokine expression. Trauma-induced CNS injury is characterized by a transient increase in proinflammatory cytokine levels that is followed by a relatively rapid restoration of baseline levels. In this instance, cytokines have a key role in the acute-phase response. Trauma related degeneration can be attributed to the initial trauma itself, since cell death and tissue necrosis occur as early as one hour after injury. On the other hand, neurodegenerative conditions like MS and ALS owe their prolonged progression to a slow and persistent increase in proinflammatory cytokines. Degenerative processes seen in these cases are more likely to be the result of direct effects of cytokines [80].

As previously noted, following any type of insult to the central nervous system, an immune response is elicited and a combination of vascular macrophages and active microglia infiltrate the lesion site. It is well documented that vascular macrophages and activated microglia synthesize and secrete many different proinflammatory cytokine and chemokine molecules [93–99]. Activated microglial cells are known to secrete a minimum of at least 20 different cytokine and chemokine molecules [100]. Some of these molecules, such as Interleukin-1 (IL-1), IL-2, IL-6, IL-15, IL-18, Interferon gamma (IFN-*γ*), and TNF-*α* are all known and documented to be proinflammatory and found to be upregulated in cases of CNS tissue damage [98, 100]. Interestingly, when these proinflammatory cytokines and chemokines, specifically IL-1, IL-2, IL-6, TNF-*α*, IFN-*γ*, were injected into normal brain tissue, a significant amount of reactive astrogliosis was observed around the injection sites [93, 94, 96]. Another study demonstrated that transforming growth factor alpha (TGF-*α*; a cytokine expressed by macrophages) and transforming growth factor beta (TGF-*β*; a cytokine expressed by both microglia and macrophages) resulted in the upregulation and synthesis of chondroitin 6-sulphate proteoglycans in brain tissue and* in vitro* cell culture experiments [99]. Recent work has identified a link between the activation of microglia and the activation of astrocytes, painting a picture of crosstalk and coregulation, where astrocytes and microglia signal to each other modulating each other's activation and post-injury activities [95, 97]. Interestingly, macrophages themselves can synthesize and either degrade or secrete CSPGs and may be a potentially significant source of CSPGs in the post-injury environment [101].

## 6. CSPG Deposition at CNS Lesions

**Figure 3:**
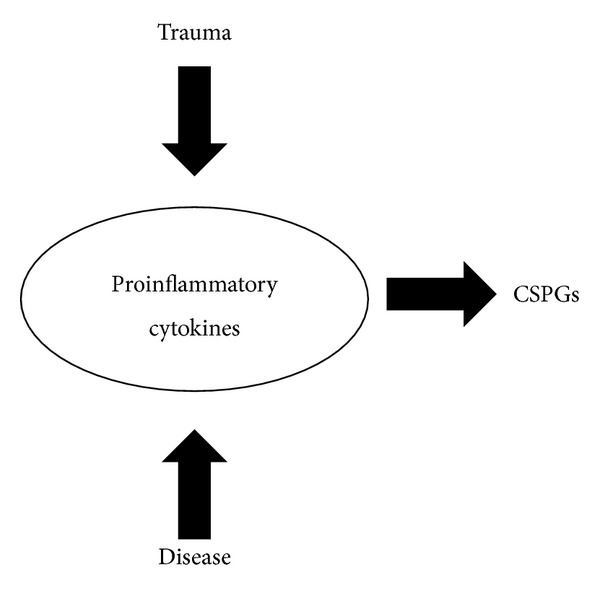
Damage to the central nervous system, either by trauma or disease processes, initiates an increase in proinflammatory cytokines, which stimulates the upregulation of CSPGs expression.

CSPGs are upregulated rapidly in the tissue surrounding a lesion site, due to the induction of reactive gliosis (see Figure 3). There are many members of the CSPG family and individual CSPGs are synthesized by different cell types and at different time points following injury. Reactive astrocytes synthesize brevican, neurocan, and phosphacan, while vascular macrophages, activated microglia, and endogenous OPCs account for the increased expression of NG2 and versican [17, 102, 103].

In traumatic lesions, CSPGs can be observed at the lesion very quickly, within the first 24 hours. However, the temporal pattern in which individual CSPG molecules are produced is different. Neurocan is the first to appear, with brevican and versican following. Their expression levels are maximal two weeks after injury, in a spinal cord injury model [103]. Peak expression of the NG2 proteoglycan occurs one week after injury, because it is expressed by infiltrating macrophages and OPCs. Keratan sulfate proteoglycans, which are also inhibitory to regeneration, are also produced by infiltrating macrophages, microglia, and OPCs as early as 3 days post-injury [103, 104]. Interestingly, expression of phosphacan is initially downregulated during the first 72 hours after lesion but slowly increases over time, reaching peak levels approximately eight weeks after injury [103]. While all members of the CSPG family are expressed following a traumatic insult to the CNS, their location in the lesion area is also variable. Neurocan is expressed close to the lesion center, and around the immediate border, in a zone from 100 *μ*m to 500 *μ*m around a spinal cord lesion. Brevican lies close to the immediate injury site as well, deposited within 300 *μ*m of the lesion border. Phosphacan and versican are expressed in a more broad range deposition, extending in a diffuse pattern as far as 600 *μ*m from the impact site [103]. Additionally, while changes in CSPG expression in tissue adjacent to the damaged tissue are well described, alterations in CSPGs expression in areas distal to lesions are also observed. In a study conducted by Andrews and colleagues [105] following a spinal contusion at the T8 level, CSPG expression was detected in both the lumbar and cervical enlargements, far away from the injury site. They discovered a strong upregulation of neurocan at the lesion epicenter and at both the lumbar and cervical enlargements. Aggrecan and brevican were initially downregulated in the lesion and was unchanged in both spinal enlargements [105]. While the total amount of NG2 protein did not change in the tissue around the lesion, there was an accumulation of NG2 in the lumbar enlargement but not the cervical. The continual and differential expression of CSPGs changes over acute and chronic times following trauma, and the changes that occur far from the site of insult maintain a broad environment inhibitory to regenerative response for many months post-injury.

In neurodegenerative lesions, the timeline of CSPG expression is not well understood, as all histopathology in humans occurs post-mortem. However, several studies have documented the presence of CSPGs in human tissue and upregulation in animal models of disease. In Alzheimer's disease, when the formation of the *β*-amyloid plaques and neurofibrillary tangles was examined, it was found that these lesions were surrounded in a shell of GFAP positive reactive astrocytes, which indicate that reactive gliosis had occurred [106]. Further examination of AD brains revealed the expression of CSPGs, heparan sulfate proteoglycans (HSPGs), and dermatan sulfate proteoglycans (DSPGs). The upregulation of CSPG expression has also been discovered in other neurodegenerative diseases such as Huntington's disease, MS, around the inclusions of Parkinson's disease, in Pick's disease, and progressive supranuclear palsy [106, 107]. The edges of active MS demyelinating lesions are rich in the CSPGs aggrecan, neurocan, and versican [108]. Further, an increase in the levels of bone morphogenic protein (BMP) has been detected in areas of demyelination. Activation of BMP stimulates increased synthesis of CSPGs from astrocytes surrounding the lesion [109]. In other forms of nontraumatic CNS injuries, such as stroke and amyotrophic lateral sclerosis, reactive astrogliosis occurs, with CSPG deposition as a result of the pathology [110, 111].

Regardless of the etiology, most CNS lesions involve an immune response, including the recruitment of vascular macrophages and activated microglial cells [10, 29, 100, 112, 113]. The cytokine and chemokine molecules expressed by these immune cell infiltrates elicit a process of reactive astrogliosis, which in turn is responsible for the upregulation of CSPG expression. While the overexpression of proteoglycans that occurs following injury is highly inhibitory to the regenerative response, it is also important to note that this process is also necessary. The upregulation of CSPGs occurs quickly to contain the tissue damage. By interfering with or preventing the formation of the glial scar, not only tissue degeneration is significantly worse, but the spread of damage into areas not initially damaged can be up to 60% greater [12–15]. This poses an interesting challenge to researchers, how to bypass or neutralize the well-known and documented inhibitory effects of CSPGs on regeneration, while not totally or permanently ablating or eliminating the formation and function of the glial scar.

## 7. Inhibitory Effect of CSPGs on Neuronal and Oligodendroglial Cells

At the molecular level,* in vitro *studies have demonstrated that CSPGs can interact with adhesion molecules expressed on various cell types [114]. When axonal growth cones come into contact with CSPGs, they collapse and retract. This is likely the reason for the abortive regenerative sprouting observed in spinal cord lesions [10, 33]. The interaction of CSPGs and neurons activates the Rho-ROCK and/or protein kinase C (PKC) intracellular signaling cascades, which inhibit process extension. It was also noted that by blocking activation of the Rho-Rock and/or PKC signalling pathways, the inhibitory effects of CSPGs could be reversed [26]. It has also been demonstrated* in vitro* that CSPGs can influence the activity of the axonal growth cone. In dorsal root ganglion cultures, the presence of CSPGs induced changes in local protein synthesis in the growth cone, with an increase in RhoA transcripts (a cytoskeletal regulator) being found locally within the growth cone after CSPG contact [115].

While CSPGs are widely accepted to be inhibitory to axonal regeneration, the inhibitory nature of individual CSPG molecules varies among the proteoglycans.* In vitro*, purified brevican has been shown to be inhibitory to both axonal attachment and growth [29, 116], while both neurocan and phosphacan have been shown to interact with neural cell adhesion molecules (N-CAM) similarly inhibiting axonal growth [29, 116]. Conversely, versican is not thought to be inhibitory to either axonal regrowth or adhesion. Axons are able to grow through deposits of versican* in vitro* and are not inhibited by the presence of purified versican [117, 118]. Certain* in vitro* studies have demonstrated that NG2 can be inhibitory to the process of axonal outgrowth, while other studies have demonstrated that NG2 is not only permissive to axonal growth but can stabilize the axon post-injury [103, 119–122]. A novel brain-derived proteoglycan Te38 is highly inhibitory to axon outgrowth [123], and it is present within the lesion site of a spinal cord injury [123, 124]. The Te38 proteoglycan could be detected for up to 4 weeks post-injury; however, the exact expression pattern for Te38 has yet to be determined.

While the inhibitory effects of CSPGs on axons have been known for some time, the effects they exert on other cell populations, such as oligodendrocytes, have only been recently considered. CSPGs exert a highly inhibitory influence on oligodendrocytes [125–127]. Studies utilizing an* in vitro* model of the glial scar with isolated OPCs revealed a significant inhibition of process outgrowth and differentiation [125]. When the OPCs processes came into contact with CSPGs, the cellular process retracted and avoided contact with the CSPG rich surface, similar to what was observed with neurons. Additional studies by other laboratories have confirmed these findings, suggesting CSPGs exert an inhibitory effect on oligodendrocytes [126, 127].

*In vivo*, CSPG expression can modulate the migration and differentiation of endogenous OPCs that are attracted to CNS lesions. Accumulation of OPCs at the edge of a lesion can be observed after spinal cord injury, in the regions distal to the lesion site [128–130]. There is somewhat conflicting evidence as to whether they can differentiate into myelinating oligodendrocytes in the area around the lesion. They may differentiate into mature cells but may not survive for longer times after injury [131, 132]. In addition to the CS-GAGs, the presence of other glycosaminoglycans like hyaluronan needs to be taken into consideration, as hyaluronan is known to modulate the behavior of oligodendrocytes. The presence of hyaluronan in active demyelinating lesions in MS and other white matter diseases can inhibit the differentiation of endogenous OPCs located near the lesions [133, 134]. Thus, the expression of glycosaminoglycans at a lesion site has effects not only on neuronal regeneration but possibly on remyelination as well.

The cellular receptors which interact with proteoglycans have only recently been identified. Potential surface receptors for proteoglycans on neurons and glial cells include the protein tyrosine phosphatase sigma (PTP*σ*). Loss of the PTP*σ* receptor by gene knockdown or inhibition of receptor activation renders neuronal process outgrowth insensitive to CSPG deposits [134]. Studies have also recently shown that the PTP*σ* receptor may also be involved with the observed inhibitory effects CSPGs exert on oligodendrocytes [127, 135]. While PTP*σ* has been observed in both neurons and oligodendrocytes, other receptors for CSPGs have been identified in neurons only, including Nogo 66 Receptor 1 (NgR1), Nogo 66 Receptor 3 (NgR3), and leukocyte common antigen receptor (LAR) [136, 137]. To date, there are no definitive reports that NgR1, NgR3, or LAR function as CSPG receptors in oligodendrocytes.

## 8. Therapeutic Modulation of CSPGs: Chondroitinase ABC

Given that CSPGs are a major barrier to repair and regeneration, a key experimental strategy for enhancing axonal regeneration and plasticity has been to modify the inhibitory extracellular environment. The bacterial enzyme chondroitinase ABC (cABC) can neutralize the inhibitory nature of the CSPG molecules and thus was tested in many injury models. cABC is an enzyme produced by the bacteria* Proteus vulgaris*, which catalyzes the removal of the glycosaminoglycan side chains from the central core protein [138]. Numerous* in vivo* studies have shown that by treating a CNS lesion site with the enzyme cABC, axonal sprouting, growth, and plasticity are significantly increased [139–147]. This is often accompanied by a significant increase in recovery of motor function, which suggests that cABC is an attractive candidate for therapeutic applications. However, to date, there is little anatomical evidence to suggest that administration of cABC has allowed for axonal regrowth over long distances. While substantial axonal growth can be observed into and even through a SCI lesion, there are no studies that demonstrate axonal regrowth to its original target. This is likely due to the slow growth rates of regenerating axons, and the short time points examined in the reported experiments. It could also be due to the fact that measured improvements in motor function result from other mechanisms, such as formation of alternative neuronal circuits, improved survival of motor neurons, as well as remyelination. The underlying explanation for the motor improvements observed after cABC administration are currently not well understood.

One critical issue with the use of cABC as a therapeutic agent is the thermal instability of the enzyme. The biological activity of cABC decreases quickly when in solution and is sensitive to temperature. A study by Tester et al. [148] demonstrated that if cABC in solution was incubated at 37°C, its enzymatic activity was significantly reduced after 3 days and totally lost by 5 days. These findings reveal that while the therapeutic ability of cABC is promising, experimental stabilization of the enzyme is likely to be needed for it to be a more effective therapeutic agent [148]. Currently, methods for administering cABC range from a constant infusion, soaking a piece of gelfoam in cABC and applying it directly to the injury site [142], to directly infusing the enzyme into the ventricles of the brain [149]. There have been several experimental approaches to stabilize the enzyme or to provide a continual supply of active enzyme at a lesion. Modification of the protein structure by amino acid substitutions has generated forms of cABC that show more stable enzymatic activity [150]. Alternatively, the gene for cABC can be inserted into a viral vector, which can be either directly introduced into a CNS lesion or introduced into cells which can then be transplanted into a lesion [151–155]. Both methods can produce sustained levels of enzyme* in vivo*, with digestion of CSPGs. To avoid the use of viral vectors, we have incorporated cABC into biodegradable nanospheres, which protects the enzymatic activity for many months [156]. When tested in an* in vivo* model of spinal cord injury, the slow release of active cABC from nanospheres resulted in a significant increase in the level of CSPG digestion at 2 weeks and 1 month after injury when compared to direct injections, as well as a significant increase in the level of axonal sprouting throughout the lesion site [157].

While cABC treatment digests CSPGs, there is evidence that following deglycanation, intact CSPGs are eventually reconstituted in the tissue. This turnover occurs approximately two-weeks following deglycanation and was demonstrated in a study by Crespo et al. [138]. In this study, a CNS lesion was inflicted to the nigrostriatal tract, followed by cABC treatment. Prepared lesion site extracts were analyzed with the 1B5 antibody to identify digested CSPGs. At 1, 4, and 7 days after lesion 1B5 labeling was clearly visible, but by 14 days after lesion 1B5 immunoreactivity was no longer present [138]. This suggests that by 2 weeks postlesion the digested CSPGs have been either reformed or cleared from the lesion site. Therefore, a continual supply of cABC within the lesion site is presumably needed until axonal outgrowth and repair of neural connections is complete. Should CSPG turnover occur before axonal outgrowth or remyelination is complete, the newly deposited CSPGs would once again create a highly inhibitory influence and halt the regeneration. However, there is clearly a need for a balance of CSPG degradation and reconstitution. While cABC treatment offers a temporary breakdown of CSPGs within the glial scar to foster axonal outgrowth, total ablation of the glial scar results in more severe tissue damage [12]. Therefore, at least the core proteins of CSPGs and perhaps the intact CSPG molecule may be needed to stabilize the spinal cord environment after CNS injury.

## 9. Postulated Mechanisms for cABC Improvements in CNS Function after Injury

While many studies claim functional recovery is a result of axonal regeneration, they fail to rule out other adaptive mechanisms that may account for the recovery of function. Such mechanisms include possible spontaneous remyelination of spared axons or the formation of alternative neural circuits [158–160]. It is essential in the field of neural regeneration research, especially when agents like cABC are tested, that the motor behavior data be correlated with neuroanatomic data to determine the cellular mechanisms underlying motor recovery. Introducing cABC into a post-injury environment rich in CSPGs, which have well-known inhibitory effects on neurons, OPCs, and possibly other cells will change their behavior in the dynamic lesion environment. Therefore, the observed recovery of function could be the collective results of multiple events not related to axonal regeneration.

Most studies using cABC have focused on the effects on neurons, documenting significant axonal sprouting and growth around a lesion after cABC treatment [139–144]. However, the extent of axonal regeneration is widely variable between studies, and no long distance regeneration is observed. While experimental treatment with cABC is frequent in the field of spinal cord injury, it has also been tested as a potential therapeutic agent in stroke and TBI research. Studies demonstrate that while the functional/behavioral recovery is mixed, in all cases of cABC administration following TBI or stroke, there are signs of anatomical reorganization, with degradation of the CSPG matrix, perineuronal nets, and evidence of axonal sprouting and growth [145–147].

It is also possible that what is sometimes characterized as axonal regeneration may actually be the sprouting of spared axons [161]. When the total percentage of CNS axons found to regenerate is compared to the results found after PNS injury, the results are striking. Under optimal conditions, on average 90% of axons are found to regenerate within the PNS [161]. However in regeneration studies examining CST axons, only 2–10% of CST axons are reported to regenerate and for relatively short distances [161]. Likewise ~7% of rubrospinal axons regenerate, at best, following experimental intervention [161]. And finally, the distance CNS axons actually grow is rather meager. As summarized by Bradbury and McMahon [161], the proportion of axons (CST axons) induced to grow longer than two spinal segments is less than 10%.

CSPGs have a strong inhibitory influence on OPC process outgrowth and differentiation, both* in vitro* and* in vivo*, and this may affect remyelination near a CNS lesion. There is significant OPC infiltration to a SCI lesion site after cABC treatment, which occurs quickly after injury [121, 124, 130, 162]. The inhibitory effects of individual CSPGs were identified using an* in vitro* assay; the strongest inhibition was observed with the mixes of CSPGs containing high levels of both neurocan and phosphacan. This is highly homologous to the CSPGs composition of the glial scar [124]. Cellular effects included stunting of cytoplasmic process outgrowth and myelin sheet formation, and impeding the migratory ability of the OPCs. It is well known that following an injury to the CNS, OPCs begin to migrate towards the site of oligodendrocyte loss and some spontaneous remyelination does occur; however, sustained remyelination of spared axons has never been well documented [22, 128].

Using a spinal cord injury model, treatment with cABC showed a significant increase in the number of OPCs found inside and around the lesion site [130]. This occurs quite quickly after injury and is independent of axonal sprouting. In the absence of cABC, OPCs migrate towards the distal edge of the lesion over the two week period. This finding mirrors that of previous studies, which show an accumulation of OPCs in the regions distal to the lesion site [128, 129]. Moreover, this also correlates to the time when the expression of CSPGs and establishment of the glial scar in the proximal area immediately adjacent to the lesion becomes maximal [10, 29, 103]. The administration of cABC immediately after injury allowed for a significant increase in overall number of OPCs as well as access into areas proximal and within the lesion. Interestingly, a large increase in OPC number was observed deep inside the lesion site [130]. The signal attracting OPCs into the lesion cavity is unknown. However, there is speculation that demyelination and myelin breakdown may be the cue. One element common to cuprizone and SCI models is the microglial activation and clearance of myelin debris [10, 29, 163]. Thus, demyelination followed by microglial activation may be recruitment signals for OPCs.

## 10. Xyloside and Other Agents

In the field of MS research, it has been demonstrated that inhibiting the synthesis of CSPGs improves the outcome of the pathology [126]. Xyloside blocks the attachment of the GAGs to the CSPG's central core protein, which is the primary inhibitory element of CSPGs. Thus, the deposition of CSPGs without GAG side chains does not create an environment that retards tissue repair. When xyloside was administered in a lysolecithin-induced model of demyelination, it not only resulted in a greatly reduced area of demyelination, but there was also a significant increase in the number of mature oligodendrocytes found within the MS plaque [126]. Due to potential side effects, xylosides may not be useful to treat SCI in humans. However, the results further support the observation that CSPGs are inhibitory to repair of the injured CNS, due to the presence of GAG side chains, and that neutralizing CSPGs allows for regenerative/reparative response to progress.

Alternatively,* in vitro *experiments have successfully targeted the enzymes responsible for the polymerization of the GAG side chains [164]. When siRNA directed against chondroitin polymerizing factor (ChPF) was introduced into astrocytes, CSPG core proteins were produced, but they were not decorated with the GAG side chain and did not pose a significant barrier to axonal growth from cerebellar granule neurons [164]. In another study, it was demonstrated that the administration of xylosyltransferase-1 (XT-1), a DNA enzyme against the GAG chain initiating enzyme, greatly reduced the presence of GAG chains, which subsequently allowed for the regeneration of microtransplanted adult sensory axons past the central core of a spinal cord stab lesion [165]. Disrupting CSPGs synthesis could provide a potentially novel therapeutic treatment paradigm, targeting assembly of the GAG chains in newly synthesized proteoglycans, rather than digestion of existing GAG chains with cABC.

## 11. Summary and Future Directions

Significant progress has been made in understanding the post-injury tissue response after CNS injury, especially the identification and characterization of molecules at the lesion site that inhibit axonal regeneration, and identifying agents that can enhance the capacity for repair. There is overwhelming evidence that following* any *insult to the CNS, traumatic or degenerative, an inflammatory reaction occurs, and the activation of microglia, astrocytes, and invasion of vascular macrophages result in an upregulation and synthesis of CSPGs (Figure 3). There is also strong evidence that CSPGs form an important protective barrier, preventing further secondary tissue damage [12–15]. However, it is also a primary reason axonal regeneration and remyelination fails following any type of injury to the CNS.

Modification of CS-GAG expression on CSPGs, while not completely ablating the CSPG molecule, is a reasonable approach to facilitating repair. One promising avenue is the administration of cABC at the site of a CNS lesion to remove the inhibitory GAG chains and neutralize the inhibitory nature of CSPGs. cABC has been tested in many injury studies and often results in an improvement in motor function. However, long distance axonal regeneration is rarely observed. In the SCI field, it is widely accepted that cABC will need to be used in conjunction with agents such as neurotrophins that can directly promote neuronal survival and stimulate axonal growth. An alternative strategy targets the new synthesis of CSPGs after injury by blocking the addition of GAG side chains. These agents also reduce the presence to the inhibitory GAG chains, but they have not been as well documented in injury models. Like cABC, it is probable that they need to be utilized in conjunction with factors that directly stimulate axonal sprouting and regrowth to maximize repair and recovery of function.

While modification of CSPG expression is an attractive therapeutic approach, there are still many questions which need to be answered. How long would an agent such as cABC be needed? Axonal growth is a slow process, which can take months in animals and longer in humans; however, cABC may only be needed at specific times to allow the regeneration process to initiate. Delivery methods are very important as well. cABC is a labile enzyme, losing activity quickly in solution. There are several choices for long term delivery including viral expression and biomaterial packaging. However, how would this be silenced once regeneration is accomplished and cABC is no longer needed? Excess cABC would destabilize perineuronal nets in uninjured tissue, which could potentially result in aberrant axonal sprouting and circuit formation as well as synaptic instability. Although there are no reports of adverse effects in animal studies, there is also the possibility that the long term presence of cABC could trigger an immune response in humans.

The most important consideration for repair of the damaged CNS is that controlling CSPG expression is only one aspect of the solution. Neurotrophic agents that directly promote neuronal survival and axonal growth are just as important, particularly for regeneration of specific neuronal populations, as are methods to direct these regenerating axons to their targets. CSPG regulation is but one important step in restoring CNS function after injury or disease.

## Figures and Tables

**Table 1:** 

Cell	Proteoglycan	CNS specific	Location	Inhibitory to axonal growth	References
Neurons	Aggrecan	No	ECM	YES	[166, 167]
	Brevican	Yes	ECM	YES	[22, 105]
	Neurocan	Yes	ECM	YES	[16, 22, 105, 168, 169]
	Phosphacan	Yes	ECM	YES	[16, 22, 105, 168, 169]

Astrocytes	Brevican	Yes	ECM	YES	[22, 105, 116]
	Neurocan	Yes	ECM	YES	[16, 22, 105, 116, 168, 169]
	Phosphacan	Yes	ECM	YES	[16, 22, 105, 116, 121, 122, 168, 169]

Activated microglial cells	KSPGs	No	TM & ECM	YES	[92, 93, 170]
	NG2	No	TM & ECM	?	[92, 108, 109, 121, 122, 171–174]

Oligodendrocyte progenitor cells	KSPGs	No	TM & ECM	YES	[92, 93, 170]
	NG2	No	TM & ECM	?	[92, 108, 109, 121, 122, 171]
	Versican (V2)	Yes	ECM	NO?	[106, 108]

Polydendrocytes	NG2	No	TM & ECM	?	[92, 108, 109, 121, 122]

Activated vascular macrophages	KSPGs	No	TM & ECM	YES	[92, 93, 170]
	NG2	No	TM & ECM	?	[92, 108, 109, 121, 122, 173, 174]

Abbreviations: TM: transmembrane; ECM: extracellular matrix.

## References

[1] Elatorai IM, Lin VW (2003). History of spinal cord medicine. *Spinal Cord Medicine: Principles & Practice*.

[2] David S, Aguayo AJ (1981). Axonal elongation into peripheral nervous system “bridges” after central nervous system injury in adult rats. *Science*.

[3] Benfey M, Aguayo AJ (1982). Extensive elongation of axons from rat brain into peripheral nerve grafts. *Nature*.

[4] Fernandes KJL, Fan DP, Tsui BJ, Cassar SL, Tetzlaff W (1999). Influence of the axotomy to cell body distance in rat rubrospinal and spinal motor neurons: differential regulation of GAP-43, Tubulins, and Neurofilament-M. *Journal of Comparative Neurology*.

[5] Hossain-Ibrahim MK, Rezajooi K, MacNally JK, Mason MRJ, Lieberman AR, Anderson PN (2006). Effects of lipopolysaccharide-induced inflammation on expression of growth-associated genes by corticospinal neurons. *BMC Neuroscience*.

[6] Mason MRJ, Lieberman AR, Anderson PN (2003). Corticospinal neurons up-regulate a range of growth-associated genes following intracortical, but not spinal, axotomy. *European Journal of Neuroscience*.

[7] Injury Prevention & Control: Traumatic Brain Injury Centers for Disease Control and Prevention. http://www.cdc.gov/TraumaticBrainInjury.

[8] SCI Information Network https://www.nscisc.uab.edu/.

[9] Young W (2002). Spinal cord contusion models. *Progress in Brain Research*.

[10] Profyris C, Cheema SS, Zang D, Azari MF, Boyle K, Petratos S (2004). Degenerative and regenerative mechanisms governing spinal cord injury. *Neurobiology of Disease*.

[11] Dawodu ST (2014). *Traumatic Brain Injury (TBI)—Definition, Epidemiology, Pathophysiology*.

[12] Faulkner JR, Herrmann JE, Woo MJ, Tansey KE, Doan NB, Sofroniew MV (2004). Reactive astrocytes protect tissue and preserve function after spinal cord injury. *The Journal of Neuroscience*.

[13] Myer DJ, Gurkoff GG, Lee SM, Hovda DA, Sofroniew MV (2006). Essential protective roles of reactive astrocytes in traumatic brain injury. *Brain*.

[14] Rolls A, Shechter R, Schwartz M (2009). The bright side of the glial scar in CNS repair. *Nature Reviews Neuroscience*.

[15] Sofroniew MV (2009). Molecular dissection of reactive astrogliosis and glial scar formation. *Trends in Neurosciences*.

[16] Bandtlow CE, Zimmermann DE (2000). Proteoglycans in the developing brain: new conceptual insights for old proteins. *Physiological Reviews*.

[17] Viapiano MS, Matthews RT (2006). From barriers to bridges: chondroitin sulfate proteoglycans in neuropathology. *Trends in Molecular Medicine*.

[18] Galtrey CM, Fawcett JW (2007). The role of chondroitin sulfate proteoglycans in regeneration and plasticity in the central nervous system. *Brain Research Reviews*.

[19] Nishiyama A, Dahlin KJ, Prince JT, Johnstone SR, Stallcup WB (1991). The primary structure of NG2, a novel membrane-spanning proteoglycan. *Journal of Cell Biology*.

[20] Lin R, Rosahl TW, Whiting PJ, Fawcett JW, Kwok JCF (2011). 6-Sulphated chondroitins have a positive influence on axonal regeneration. *PLoS ONE*.

[21] Wang H, Katagiri Y, McCann TE (2008). Chondroitin-4-sulfation negatively regulates axonal guidance and growth. *Journal of Cell Science*.

[22] Yi JH, Katagiri Y, Susarla B, Figge D, Symes AJ, Geller HM (2012). Alterations in sulfated chondroitin glycosaminoglycans following controlled cortical impact injury in mice. *The Journal of Comparative Neurology*.

[23] Snow DM, Lemmon V, Carrino DA, Caplan AI, Silver J (1990). Sulfated proteoglycans in astroglial barriers inhibit neurite outgrowth *in vitro*. *Experimental Neurology*.

[24] Clement AM, Nadanaka S, Masayama K, Mandl C, Sugahara K, Faissner A (1998). The DSD-1 carbohydrate epitope depends on sulfation, correlates with chondroitin sulfate D motifs, and is sufficient to promote neurite outgrowth. *The Journal of Biological Chemistry*.

[25] Clement AM, Sugahara K, Faissner A (1999). Chondroitin sulfate E promotes neurite outgrowth of rat embryonic day 18 hippocampal neurons. *Neuroscience Letters*.

[26] Busch SA, Silver J (2007). The role of extracellular matrix in CNS regeneration. *Current Opinion in Neurobiology*.

[27] Rhodes KE, Fawcett JW (2004). Chondroitin sulphate proteoglycans: preventing plasticity or protecting the CNS?. *Journal of Anatomy*.

[28] Thuret S, Moon LDF, Gage FH (2006). Therapeutic interventions after spinal cord injury. *Nature Reviews Neuroscience*.

[29] Fawcett JW, Asher RA (1999). The glial scar and central nervous system repair. *Brain Research Bulletin*.

[30] Norenberg MD, Smith J, Marcillo A (2004). The pathology of human spinal cord injury: defining the Problems. *Journal of Neurotrauma*.

[31] Stoll G, Trapp BD, Griffin JW (1989). Macrophage function during Wallerian degeneration of rat optic nerve: clearance of degenerating myelin and Ia expression. *Journal of Neuroscience*.

[32] Perry VH, Brown MC, Gordon S (1987). The macrophage response to central and peripheral nerve injury: a possible role for macrophages in regeneration. *Journal of Experimental Medicine*.

[33] Fleming JC, Norenberg MD, Ramsay DA (2006). The cellular inflammatory response in human spinal cords after injury. *Brain*.

[34] Kigerl KA, Gensel JC, Ankeny DP, Alexander JK, Donnelly DJ, Popovich PG (2009). Identification of two distinct macrophage subsets with divergent effects causing either neurotoxicity or regeneration in the injured mouse spinal cord. *Journal of Neuroscience*.

[35] Barron KD (1995). The microglial cell. A historical review. *Journal of the Neurological Sciences*.

[36] Aloisi F (2001). Immune function of microglia. *Glia*.

[37] Rock RB, Gekker G, Hu S (2004). Role of microglia in central nervous system infections. *Clinical Microbiology Reviews*.

[38] Popovich PG, Wei P, Stokes BT (1997). Cellular inflammatory response after spinal cord injury in Sprague-Dawley and Lewis rats. *Journal of Comparative Neurology*.

[39] Bandtlow CE, Schmidt MF, Hassinger TD, Schwab ME, Kater SB (1993). Role of intracellular calcium in NI-35-evoked collapse of neuronal growth cones. *Science*.

[40] Yiu G, He Z (2006). Glial inhibition of CNS axon regeneration. *Nature Reviews Neuroscience*.

[41] Masters CL, Beyreuther K (1987). Neuronal origin of cerebral amyloidogenic proteins: their role in Alzheimer's disease and unconventional virus diseases of the nervous system. *Ciba Foundation Symposium*.

[42] Braak H, Braak E, Strothjohann M (1994). Abnormally phosphorylated tau protein related to the formation of neurofibrillary tangles and neuropil threads in the cerebral cortex of sheep and goat. *Neuroscience Letters*.

[43] Coraci IS, Husemann J, Berman JW (2002). CD36, a class B scavenger receptor, is expressed on microglia in Alzheimer's disease brains and can mediate production of reactive oxygen species in response to *β*-amyloid fibrils. *The American Journal of Pathology*.

[44] Jarrett JT, Berger EP, Lansbury PT (1993). The C-terminus of the *β* protein is critical in amyloidogenesis. *Annals of the New York Academy of Sciences*.

[45] Itagaki S, McGeer PL, Akiyama H, Zhu S, Selkoe D (1989). Relationship of microglia and astrocytes to amyloid deposits of Alzheimer disease. *Journal of Neuroimmunology*.

[46] McGeer PL, Akiyama H, Itagaki S, McGeer EG (1989). Activation of the classical complement pathway in brain tissue of Alzheimer patients. *Neuroscience Letters*.

[47] McGeer PL, Akiyama H, Itagaki S, McGeer EG (1989). Immune system response in Alzheimer's disease. *Canadian Journal of Neurological Sciences*.

[48] McGeer PL, McGeer EG (2000). Autotoxicity and Alzheimer disease. *Archives of Neurology*.

[49] Akiyama H, Barger S, Barnum S (2000). Inflammation and Alzheimer's disease. *Neurobiology of Aging*.

[50] Gupta A, Pansari K (2003). Inflammation and Alzheimer's disease. *International Journal of Clinical Practice*.

[51] McGeer PL, McGeer EG (2001). Inflammation, autotoxicity and Alzheimer disease. *Neurobiology of Aging*.

[52] McGeer PL, McGeer EG (2002). Local neuroinflammation and the progression of Alzheimer's disease. *Journal of NeuroVirology*.

[53] McGeer PL, McGeer EG, Yasojima K (2000). Alzheimer disease and neuroinflammation. *Journal of Neural Transmission, Supplement*.

[54] Teismann P, Tieu K, Choi D (2003). Cyclooxygenase-2 is instrumental in Parkinson's disease neurodegeneration. *Proceedings of the National Academy of Sciences of the United States of America*.

[55] Fahn S (2003). Description of Parkinson's disease as a clinical syndrome. *Annals of the New York Academy of Sciences*.

[56] Hunot S, Hirsch EC (2003). Neuroinflammatory processes in Parkinson's disease. *Annals of Neurology*.

[57] Orr CF, Rowe DB, Halliday GM (2002). An inflammatory review of Parkinson’s disease. *Progress in Neurobiology*.

[58] Wu DC, Jackson-Lewis V, Vila M (2002). Blockade of microglial activation is neuroprotective in the 1-methyl-4-phenyl-1,2,3,6-tetrahydropyridine mouse model of Parkinson disease. *Journal of Neuroscience*.

[59] Liu B, Du L, Hong J (2000). Naloxone protects rat dopaminergic neurons against inflammatory damage through inhibition of microglia activation and superoxide generation. *Journal of Pharmacology and Experimental Therapeutics*.

[60] Grünblatt E, Mandel S, Youdim MBH (2000). Neuroprotective strategies in Parkinson's disease using the models of 6-hydroxydopamine and MPTP. *Annals of the New York Academy of Sciences*.

[61] Liberatore GT, Jackson-Lewis V, Vukosavic S (1999). Inducible nitric oxide synthase stimulates dopaminergic neurodegeneration in the MPTP model of Parkinson disease. *Nature Medicine*.

[62] McGeer PL, Itagaki S, Tago H, McGeer EG (1988). Occurrence of HLA-DR reactive microglia in Alzheimer's disease. *Annals of the New York Academy of Sciences*.

[63] Mogi M, Harada M, Kondo T, Riederer P, Nagatsu T (1995). Brain *β*
_2_-microglobulin levels are elevated in the striatum in Parkinson's diseaselevels are elevated in the striatum in Parkinson's disease. *Journal of Neural Transmission: Parkinson's Disease and Dementia Section*.

[64] Rodríguez M, Barroso-Chinea P, Abdala P, Obeso J, González-Hernández T (2001). Dopamine cell degeneration induced by intraventricular administration of 6-hydroxydopamine in the rat: similarities with cell loss in Parkinson's disease. *Experimental Neurology*.

[65] Martino G, Clementi E, Brambilla E (1994). *γ* interferon activates a previously undescribed Ca^2+^ influx in T lymphocytes from patients with multiple sclerosis. *Proceedings of the National Academy of Sciences of the United States of America*.

[66] Correale J, De los Milagros Bassani Molinas M (2002). Oligoclonal bands and antibody responses in multiple sclerosis. *Journal of Neurology*.

[67] Lassmann H, Brück W, Lucchinetti C (2001). Heterogeneity of multiple sclerosis pathogenesis: implications for diagnosis and therapy. *Trends in Molecular Medicine*.

[68] Proescholdt MA, Jacobson S, Tresser N, Oldfield EH, Merrill MJ (2002). Vascular endothelial growth factor is expressed in multiple sclerosis plaques and can induce inflammatory lesions in experimental allergic encephalomyelitis rats. *Journal of Neuropathology & Experimental Neurology*.

[69] Selmaj KW, Farooq M, Norton WT, Raine CS, Brosnan CF (1990). Proliferation of astrocytes in vitro in response to cytokines. A primary role for tumor necrosis factor. *Journal of Immunology*.

[70] Aloisi F, Giampaolo A, Russo G, Peschle C, Levi G (1992). Developmental appearance, antigenic profile, and proliferation of glial cells of the human embryonic spinal cord: an immunocytochemical study using dissociated cultured cells. *Glia*.

[71] Frei K, Nohava K, Malipiero UV, Schwerdel C, Fontana A (1992). Production of macrophage colony-stimulating factor by astrocytes and brain macrophages. *Journal of Neuroimmunology*.

[72] Giulian D, Ingeman JF (1988). Colony-stimulating factors as promoters of ameboid microglia. *Journal of Neuroscience*.

[73] Wong PC, Cai H, Borchelt DR, Price DL (2002). Genetically engineered mouse models of neurodegenerative diseases. *Nature Neuroscience*.

[74] Torreilles F, Salman-Tabcheh S, Guérin M, Torreilles J (1999). Neurodegenerative disorders: the role of peroxynitrite. *Brain Research Reviews*.

[75] McGeer PL, McGeer EG (2002). Inflammatory processes in amyotrophic lateral sclerosis. *Muscle and Nerve*.

[76] Hensley K, Floyd RA, Gordon B (2002). Temporal patterns of cytokine and apoptosis-related gene expression in spinal cords of the G93A-SOD1 mouse model of amyotrophic lateral sclerosis. *Journal of neurochemistry*.

[77] Akiyama H, McGeer PL (1990). Brain microglia constitutively express *β*-2 integrins. *Journal of Neuroimmunology*.

[78] Kawamata T, Akiyama H, Yamada T, McGeer PL (1992). Immunologic reactions in amyotrophic lateral sclerosis brain and spinal cord tissue. *The American Journal of Pathology*.

[79] Troost D, van den Oord JJ, Vianney de Jong JMB (1990). Immunohistochemical characterization of the inflammatory infiltrate in amyotrophic lateral sclerosis. *Neuropathology and Applied Neurobiology*.

[80] Popovich PG, Stokes BT, Whitacre CC (1996). Concept of autoimmunity following spinal cord injury: possible roles for T lymphocytes in the traumatized central nervous system. *Journal of Neuroscience Research*.

[81] Gasche Y, Soccal PM, Kanemitsu M, Copin J (2006). Matrix metalloproteinases and diseases of the central nervous system with a special emphasis on ischemic brain. *Frontiers in Bioscience*.

[82] Weiss N, Miller F, Cazaubon S, Couraud PO (2009). The blood-brain barrier in brain homeostasis and neurological diseases. *Biochimica et Biophysica Acta*.

[83] Obermeier B, Daneman R, Ransohoff RM (2013). Development maintenance and disruption of the blood-brain barrier. *Nature Medicine*.

[84] Popescu BO, Toescu EC, Popescu LM (2009). Blood-brain barrier alterations in ageing and dementia. *Journal of the Neurological Sciences*.

[85] Friedlander RM, Yuan J (1998). ICE, neuronal apoptosis and neurodegeneration. *Cell Death and Differentiation*.

[86] Kim G, Xu J, Song S (2001). Tumor necrosis factor receptor deletion reduces nuclear factor-*κ*B activation, cellular inhibitor of apoptosis protein 2 expression, and functional recovery after traumatic spinal cord injury. *Journal of Neuroscience*.

[87] Selmaj K, Walczak A, Mycko M, Berkowicz T, Kohno T, Raine CS (1998). Suppression of experimental autoimmune encephalomyelitis with a TNF binding protein (TNFbp) correlates with down-regulation of VCAM-1/VLA-4. *European Journal of Immunology*.

[88] Selmaj KW (2000). Tumour necrosis factor and anti-tumour necrosis factor approach to inflammatory demyelinating diseases of the central nervous system. *Annals of the Rheumatic Diseases*.

[89] Streit WJ, Walter SA, Pennell NA (1999). Reactive microgliosis. *Progress in Neurobiology*.

[90] Tuna M, Polat S, Erman T (2001). Effect of anti-rat interleukin-6 antibody after spinal cord injury in the rat: inducible nitric oxide synthase expression, sodium- and potassium-activated, magnesium-dependent adenosine-5′-triphosphatase and superoxide dismutase activation, and ultrastructural changes. *Journal of Neurosurgery*.

[91] Kahl KG, Kruse N, Toyka KV, Rieckmann P (2002). Serial analysis of cytokine mRNA profiles in whole blood samples from patients with early multiple sclerosis. *Journal of the Neurological Sciences*.

[92] Popovich PG, Guan Z, Wei P, Huitinga I, Van Rooijen N, Stokes BT (1999). Depletion of hematogenous macrophages promotes partial hindlimb recovery and neuroanatomical repair after experimental spinal cord injury. *Experimental Neurology*.

[93] Balasingam V, Tejada-Berges T, Wright E, Bouckova R, Yong VW (1994). Reactive astrogliosis in the neonatal mouse brain and its modulation by cytokines. *Journal of Neuroscience*.

[94] Giulian D, Woodward J, Young DG, Krebs JF, Lachman LB (1988). Interleukin-1 injected into mammalian brain stimulates astrogliosis and neovascularization. *Journal of Neuroscience*.

[95] Shechter R, Raposo C, London A, Sagi I, Schwartz M (2011). The glial scar-monocyte interplay: a pivotal resolution phase in spinal cord repair. *PLoS ONE*.

[96] Yong VW, Moumdjian R, Young FP (1991). *γ*-Interferon promotes proliferation of adult human astrocytes *in vitro* and reactive gliosis in the adult mouse brain *in vivo*. *Proceedings of the National Academy of Sciences of the United States of America*.

[97] Liu W, Tang Y, Feng J (2011). Cross talk between activation of microglia and astrocytes in pathological conditions in the central nervous system. *Life Sciences*.

[98] Smith JA, Das A, Ray SK, Banik NL (2012). Role of pro-inflammatory cytokines released from microglia in neurodegenerative diseases. *Brain Research Bulletin*.

[99] Properzi F, Carulli D, Asher RA (2005). Chondroitin 6-sulphate synthesis is up-regulated in injured CNS, induced by injury-related cytokines and enhanced in axon-growth inhibitory glia. *European Journal of Neuroscience*.

[100] Hanisch U (2002). Microglia as a source and target of cytokines. *GLIA*.

[101] Uhlin-Hansen L, Wik T, Kjellen L, Berg E, Forsdahl F, Kolset SO (1993). Proteoglycan metabolism in normal and inflammatory human macrophages. *Blood*.

[102] Asher RA, Morgenstern DA, Shearer MC, Adcock KH, Pesheva P, Fawcett JW (2002). Versican is upregulated in CNS injury and is a product of oligodendrocyte lineage cells. *Journal of Neuroscience*.

[103] Jones LL, Margolis RU, Tuszynski MH (2003). The chondroitin sulfate proteoglycans neurocan, brevican, phosphacan, and versican are differentially regulated following spinal cord injury. *Experimental Neurology*.

[104] Imagama S, Sakamoto K, Tauchi R (2011). Keratan sulfate restricts neural plasticity after spinal cord injury. *Journal of Neuroscience*.

[105] Andrews EM, Richards RJ, Yin FQ, Viapiano MS, Jakeman LB (2012). Alterations in chondroitin sulfate proteoglycan expression occur both at and far from the site of spinal contusion injury. *Experimental Neurology*.

[106] McGeer PL, McGeer EG (1995). The inflammatory response system of brain: implications for therapy of Alzheimer and other neurodegenerative diseases. *Brain Research Reviews*.

[107] Bonneh-Barkay D, Wiley CA (2009). Brain extracellular matrix in neurodegeneration. *Brain Pathology*.

[108] Sobel RA, Ahmed AS (2001). White matter extracellular matrix chondroitin sulfate/dermatan sulfate proteoglycans in multiple sclerosis. *Journal of Neuropathology and Experimental Neurology*.

[109] Fuller ML, DeChant AK, Rothstein B (2007). Bone morphogenetic proteins promote gliosis in demyelinating spinal cord lesions. *Annals of Neurology*.

[110] Barreto G, White RE, Ouyang Y, Xu L, Giffard RG (2011). Astrocytes: Targets for neuroprotection in stroke. *Central Nervous System Agents in Medicinal Chemistry*.

[111] Mizuno H, Warita H, Aoki M, Itoyama Y (2008). Accumulation of chondroitin sulfate proteoglycans in the microenvironment of spinal motor neurons in amyotrophic lateral sclerosis transgenic rats. *Journal of Neuroscience Research*.

[112] Tansey MG, McCoy MK, Frank-Cannon TC (2007). Neuroinflammatory mechanisms in Parkinson's disease: potential environmental triggers, pathways, and targets for early therapeutic intervention. *Experimental Neurology*.

[113] Town T, Nikolic V, Tan J (2005). The microglial “activation” continuum: From innate to adaptive responses. *Journal of Neuroinflammation*.

[114] Friedlander DR, Milev P, Karthikeyan L, Margolis RK, Margolis RU, Grumet M (1994). The neuronal chondroitin sulfate proteoglycan neurocan binds to the neural cell adhesion molecules Ng-CAM/L1/NILE and N-CAM, and inhibits neuronal adhesion and neurite outgrowth. *Journal of Cell Biology*.

[115] Walker BA, Ji S, Jaffrey SR (2012). Intra-axonal translation of RhoA promotes axon growth inhibition by CSPG. *Journal of Neuroscience*.

[116] Dow KE, Wang W (1998). Cell biology of astrocyte proteoglycans. *Cellular and Molecular Life Sciences*.

[117] Braunewell K-H, Pesheva P, McCarthy JB, Furcht LT, Schmitz B, Schachner M (1995). Functional involvement of sciatic nerve-derived versican and decorin-like molecules and other chondroitin sulphate proteoglycans in ECM-mediated cell adhesion and neurite outgrowth. *European Journal of Neuroscience*.

[118] Fidler PS, Schuette K, Asher RA (1999). Comparing astrocytic cell lines that are inhibitory or permissive for axon growth: the major axon-inhibitory proteoglycan is NG2. *Journal of Neuroscience*.

[119] Tan AM, Zhang W, Levine JM (2005). NG2: a component of the glial scar that inhibits axon growth. *Journal of Anatomy*.

[120] McTigue DM, Tripathi R, Wei P (2006). NG2 colocalizes with axons and is expressed by a mixed cell population in spinal cord lesions. *Journal of Neuropathology and Experimental Neurology*.

[121] Yang Z, Suzuki R, Daniels SB, Brunquell CB, Sala CJ, Nishiyama A (2006). NG2 glial cells provide a favorable substrate for growing axons. *Journal of Neuroscience*.

[122] Busch SA, Horn KP, Cuascut FX (2010). Adult NG2+cells are permissive to neurite outgrowth and stabilize sensory axons during macrophage-induced axonal dieback after spinal cord injury. *Journal of Neuroscience*.

[123] Henke-Fahle S, Wild K, Sierra A, Monnier PP (2001). Characterization of a new brain-derived proteoglycan inhibiting retinal ganglion cell axon outgrowth. *Molecular and Cellular Neuroscience*.

[124] Monnier PP, Sierra A, Schwab JM, Henke-Fahle S, Mueller BK (2003). The Rho/ROCK pathway mediates neurite growth-inhibitory activity associated with the chondroitin sulfate proteoglycans of the CNS glial scar. *Molecular and Cellular Neuroscience*.

[125] Siebert JR, Osterhout DJ (2011). The inhibitory effects of chondroitin sulfate proteoglycans on oligodendrocytes. *Journal of Neurochemistry*.

[126] Lau LW, Keough MB, Haylock-Jacobs S (2012). Chondroitin sulfate proteoglycans in demyelinated lesions impair remyelination. *Annals of Neurology*.

[127] Pendleton JC, Shamblott MJ, Gary DS (2013). Chondroitin sulfate proteoglycans inhibit oligodendrocyte myelination through PTPσ. *Experimental Neurology*.

[128] Levine JM, Reynolds R, Fawcett JW (2001). The oligodendrocyte precursor cell in health and disease. *Trends in Neurosciences*.

[129] Tripathi RB, McTigue DM (2008). Chronically increased ciliary neurotrophic factor and fibroblast growth factor-2 expression after spinal contusion in rats. *Journal of Comparative Neurology*.

[130] Siebert JR, Stelzner DJ, Osterhout DJ (2011). Chondroitinase treatment following spinal contusion injury increases migration of oligodendrocyte progenitor cells. *Experimental Neurology*.

[131] Crowe MJ, Bresnahan JC, Shuman Sl, Masters JN, Beattie MS (1997). Apoptosis and delayed degeneration after spinal cord injury in rats and monkeys. *Nature Medicine*.

[132] Bramlett HM, Dietrich WD (2007). Progressive damage after brain and spinal cord injury: pathomechanisms and treatment strategies. *Progress in Brain Research*.

[133] Back SA, Tuohy TMF, Chen H (2005). Hyaluronan accumulates in demyelinated lesions and inhibits oligodendrocyte progenitor maturation. *Nature Medicine*.

[134] Bugiani M, Postma N, Polder E (2013). Hyaluronan accumulation and arrested oligodendrocyte progenitor maturation in vanishing white matter disease. *Brain*.

[135] Harlow DE, Macklin WB (2014). Inhibitors of remyelination: ECM changes, SPGs and PTPs. *Experimental Neurology*.

[136] Fisher D, Xing B, Dill J (2011). Leukocyte common antigen-related phosphatase is a functional receptor for chondroitin sulfate proteoglycan axon growth inhibitors. *Journal of Neuroscience*.

[137] Dickendesher TL, Baldwin KT, Mironova YA (2012). NgR1 and NgR3 are receptors for chondroitin sulfate proteoglycans. *Nature Neuroscience*.

[138] Crespo D, Asher RA, Lin R, Rhodes KE, Fawcett JW (2007). How does chondroitinase promote functional recovery in the damaged CNS?. *Experimental Neurology*.

[139] Zuo J, Neubauer D, Dyess K, Ferguson TA, Muir D (1998). Degradation of chondroitin sulfate proteoglycan enhances the neurite- promoting potential of spinal cord tissue. *Experimental Neurology*.

[140] Yick L, Wu W, So K, Yip HK, Shum DK (2000). Chondroitinase ABC promotes axonal regeneration of Clarke's neurons after spinal cord injury. *NeuroReport*.

[141] Bradbury EJ, Moon LDF, Popat RJ (2002). Chondroitinase ABC promotes functional recovery after spinal cord injury. *Nature*.

[142] Yick L, Cheung P, So K, Wu W (2003). Axonal regeneration of Clarke's neurons beyond the spinal cord injury scar after treatment with chondroitinase ABC. *Experimental Neurology*.

[143] Barritt AW, Davies M, Marchand F (2006). Chondroitinase ABC promotes sprouting of intact and injured spinal systems after spinal cord injury. *Journal of Neuroscience*.

[144] Tom VJ, Kadakia R, Santi L, Houlé JD (2009). Administration of chondroitinase ABC rostral or caudal to a spinal cord injury site promotes anatomical but not functional plasticity. *Journal of Neurotrauma*.

[145] Soleman S, Yip PK, Duricki DA, Moon LDF (2012). Delayed treatment with chondroitinase ABC promotes sensorimotor recovery and plasticity after stroke in aged rats. *Brain*.

[146] Hill JJ, Jin K, Mao XO, Xie L, Greenberg DA (2012). Intracerebral chondroitinase ABC and heparan sulfate proteoglycan glypican improve outcome from chronic stroke in rats. *Proceedings of the National Academy of Sciences of the United States of America*.

[147] Harris NG, Mironova YA, Hovda DA, Sutton RL (2010). Chondroitinase ABC enhances pericontusion axonal sprouting but does not confer robust improvements in behavioral recovery. *Journal of Neurotrauma*.

[148] Tester NJ, Plaas AH, Howland DR (2007). Effect of body temperature on chondroitinase ABC's ability to cleave chondroitin sulfate glycosaminoglycans. *Journal of Neuroscience Research*.

[149] García-Alías G, Lin R, Akrimi SF, Story D, Bradbury EJ, Fawcett JW (2008). Therapeutic time window for the application of chondroitinase ABC after spinal cord injury. *Experimental Neurology*.

[150] Nazari-Robati M, Khajeh K, Aminian M, Mollania N, Golestani A (2013). Enhancement of thermal stability of chondroitinase ABC i by site-directed mutagenesis: an insight from Ramachandran plot. *Biochimica et Biophysica Acta - Proteins and Proteomics*.

[151] Curinga GM, Snow DM, Mashburn C (2007). Mammalian-produced chondroitinase AC mitigates axon inhibition by chondroitin sulfate proteoglycans. *Journal of Neurochemistry*.

[152] Jin Y, Ketschek A, Jiang Z, Smith G, Fischer I (2011). Chondroitinase activity can be transduced by a lentiviral vector *in vitro* and *in vivo*. *Journal of Neuroscience Methods*.

[153] Zhao R, Muir EM, Alves JN (2011). Lentiviral vectors express chondroitinase ABC in cortical projections and promote sprouting of injured corticospinal axons. *Journal of Neuroscience Methods*.

[154] Kanno H, Pressman Y, Moody A (2014). Combination of engineered Schwann cell grafts to secrete neurotrophin and chondroitinase promotes axonal regeneration and locomotion after spinal cord injury. *The Journal of Neuroscience*.

[155] Bartus K, James ND, Didangelos A (2014). Large-scale chondroitin sulfate proteoglycan digestion with chondroitinase gene therapy leads to reduced pathology and modulates macrophage phenotype following spinal cord contusion injury. *Journal of Neuroscience*.

[156] Lannu JD, Choi R, Au A Chondroitinase activity is protected for extended times when incorporated into nanospheres for time release delivery.

[157] Osterhout DJ, Garma PD, Au A Chondroitinase release from nanospheres induces axonal sprouting after spinal cord injury.

[158] Bareyre FM, Kerschensteiner M, Raineteau O, Mettenleiter TC, Weinmann O, Schwab ME (2004). The injured spinal cord spontaneously forms a new intraspinal circuit in adult rats. *Nature Neuroscience*.

[159] Maier IC, Schwab ME (2006). Sprouting, regeneration, and circuit formation in the injured spinal cord: factors and activity. *Nature Medicine*.

[160] Courtine G, Song B, Roy RR (2008). Recovery of supraspinal control of stepping via indirect propriospinal relay connections after spinal cord injury. *Nature Medicine*.

[161] Bradbury EJ, McMahon SB (2006). Spinal cord repair strategies: why do they work?. *Nature Reviews Neuroscience*.

[162] Karimi-Abdolrezaee S, Schut D, Wang J, Fehlings MG (2012). Chondroitinase and growth factors enhance activation and oligodendrocyte differentiation of endogenous neural precursor cells after spinal cord injury. *PLoS ONE*.

[163] Matsushima GK, Morell P (2001). The neurotoxicant, cuprizone, as a model to study demyelination and remyelination in the central nervous system. *Brain Pathology*.

[164] Laabs TL, Wang H, Katagiri Y, McCann T, Fawcett JW, Geller HM (2007). Inhibiting glycosaminoglycan chain polymerization decreases the inhibitory activity of astrocyte-derived chondroitin sulfate proteoglycans. *Journal of Neuroscience*.

[165] Grimpe B, Silver J (2004). A novel DNA enzyme reduces glycosaminoglycan chains in the glial scar and allows microtransplanted dorsal root ganglia axons to regenerate beyond lesions in the spinal cord. *Journal of Neuroscience*.

[166] Lemons ML, Sandy JD, Anderson DK, Howland DR (2003). Intact aggrecan and chondroitin sulfate-depleted aggrecan core glycoprotein inhibit axon growth in the adult rat spinal cord. *Experimental Neurology*.

[167] Silver J, Miller JH (2004). Regeneration beyond the glial scar. *Nature Reviews Neuroscience*.

[168] Grumet M, Milev P, Sakurai T (1994). Interactions with tenascin and differential effects on cell adhesion of neurocan and phosphacan, two major chondroitin sulfate proteoglycans of nervous tissue. *The Journal of Biological Chemistry*.

[169] Margolis RK, Rauch UWE, Maurel P, Margolis RU (1996). Neurocan and phosphacan: two major nervous tissue-specific chondroitin sulfate proteoglycans. *Perspectives on Developmental Neurobiology*.

[170] Jones LL, Tuszynski MH (2002). Spinal cord injury elicits expression of keratan sulfate proteoglycans by macrophages, reactive microglia, and oligodendrocyte progenitors. *The Journal of Neuroscience*.

[171] Jones LL, Yamaguchi Y, Stallcup WB, Tuszynski MH (2002). NG2 is a major chondroitin sulfate proteoglycan produced after spinal cord injury and is expressed by macrophages and oligodendrocyte progenitors. *Journal of Neuroscience*.

[172] Zhu L, Lu J, Tay SSW, Jiang H, He BP (2010). Induced NG2 expressing microglia in the facial motor nucleus after facial nerve axotomy. *Neuroscience*.

[173] Bu J, Akhtar N, Nishiyama A (2001). Transient expression of the NG2 proteoglycan by a subpopulation of activated macrophages in an excitotoxic hippocampal lesion. *GLIA*.

[174] Zhu L, Xiang P, Guo K (2012). Microglia/monocytes with NG2 expression have no phagocytic function in the cortex after LPS focal injection into the rat brain. *GLIA*.

